# Prevalence and molecular typing of carbapenem-resistant Enterobacterales in a secondary-care hospital in Jazan region, Saudi Arabia

**DOI:** 10.1016/j.nmni.2026.101825

**Published:** 2026-08-13

**Authors:** Hanan Alshafie, Fathima Tarannum, Mousa Ayyashi, Nawal Yaqoub, Maryam Hazazi, Sultan Masmali, Durga Prasad Dommeti, Halima Babgi

**Affiliations:** a Department of Medical Microbiology Laboratory, Prince Mohammed bin Nasser Hospital, Jazan Health Cluster, Jazan, Saudi Arabia; b Department of Laboratory, Prince Mohammed bin Nasser Hospital, Jazan Health Cluster, Jazan, Saudi Arabia; c Department of Population Health Management, Jazan Health Cluster, Jazan, Saudi Arabia; d Department of Laboratory, Jazan Specialized Hospital, Jazan Health Cluster, Jazan, Saudi Arabia; e Department of Quality, Jazan Specialized Hospital, Jazan Health Cluster, Jazan, Saudi Arabia

**Keywords:** Carbapenem-resistant enterobacterales, Prevalence, Carbapenemase, Antimicrobial resistance, Saudi Arabia

## Abstract

**Objectives:**

The aim of this study was to estimate the prevalence of carbapenem-resistant Enterobacterales (CRE) and characterize the distribution of carbapenemase genes among Enterobacterales isolates in the medical microbiology laboratory of Prince Mohammed Bin Nasser Hospital, Jazan, Saudi Arabia.

**Methods:**

The current cross-sectional study was conducted in a secondary-care center in Jazan, Saudi Arabia, and included consecutive Enterobacterales isolates from clinical specimens of hospitalized patients from June 2023 to April 2024. Demographic data and clinically relevant information were retrieved from electronic medical records. The statistical analyses were performed using the software R and IBM SPSS Statistics.

**Results:**

A total of 260 Enterobacterales isolates were studied; of these, 26 (10%) fulfilled the criteria for CRE. In the subpopulation of CRE, *Klebsiella pneumoniae* was the most common (81%), followed by *Enterobacter cloacae* complex (11%), *Escherichia coli* (4%), and *Klebsiella oxytoca* (4%). The most common resistant CRE gene was NDM (58%), followed by a combination of OXA48 and NDM (23%), and then OXA48 (19%). Statistically significant associations existed for admission to a critical care unit, the presence of a central line, and the use of a urinary catheter with the isolation of CRE.

**Conclusion:**

In this hospital-based cohort, CRE were commonly found among Enterobacterales isolates, mainly *K. pneumoniae*, and were predominantly driven by the NDM and OXA-48-type carbapenemases. Associations between CRE isolation and exposure to critical care and invasive devices suggest strengthened surveillance, device-related infection-prevention measures, and antimicrobial stewardship should be emphasized in this setting, with a focus on high-risk inpatients.

## 1 Introduction

**Fig. 1:**
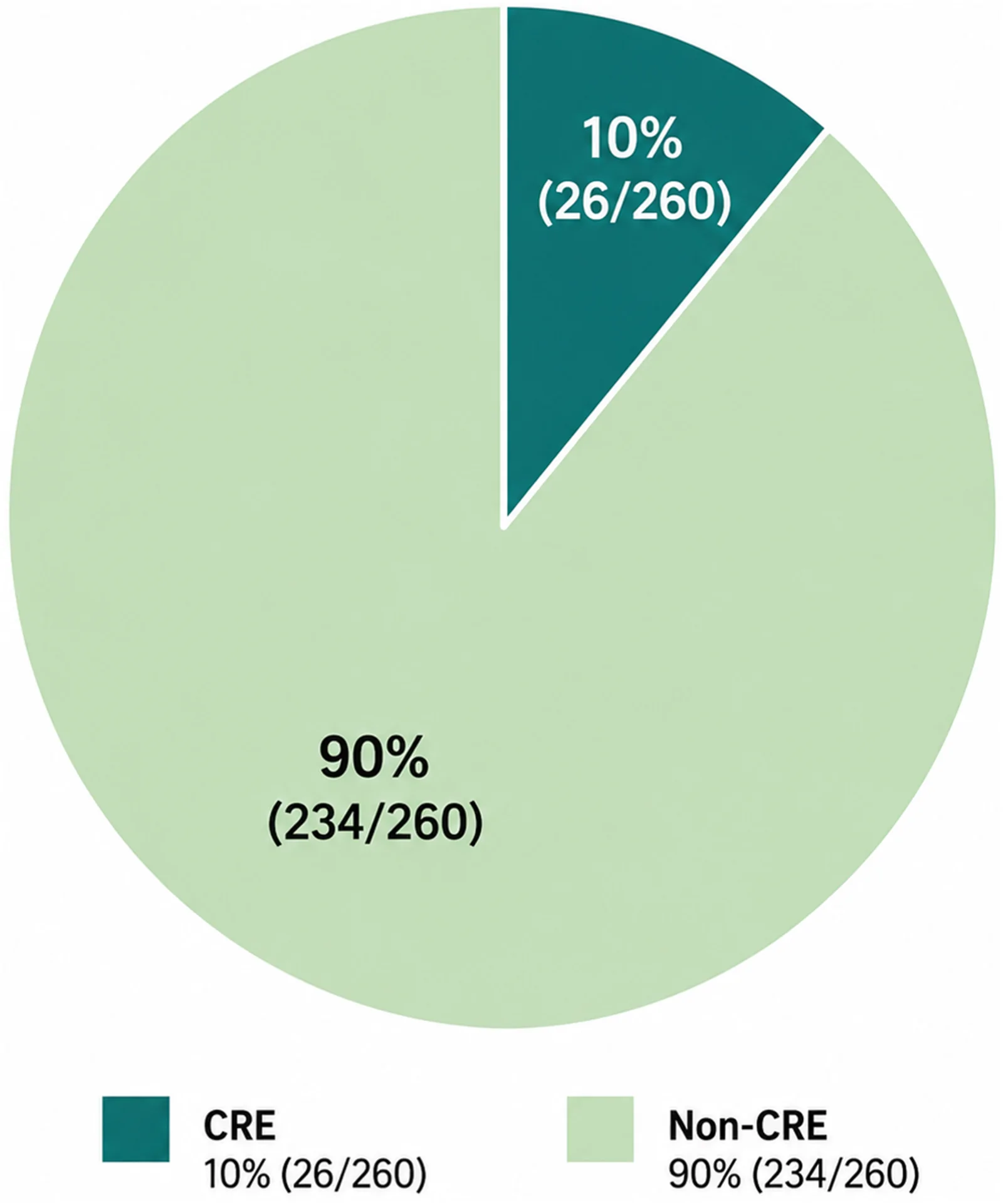
Prevalence of CRE among Enterobacterales isolates.

**Fig. 2:**
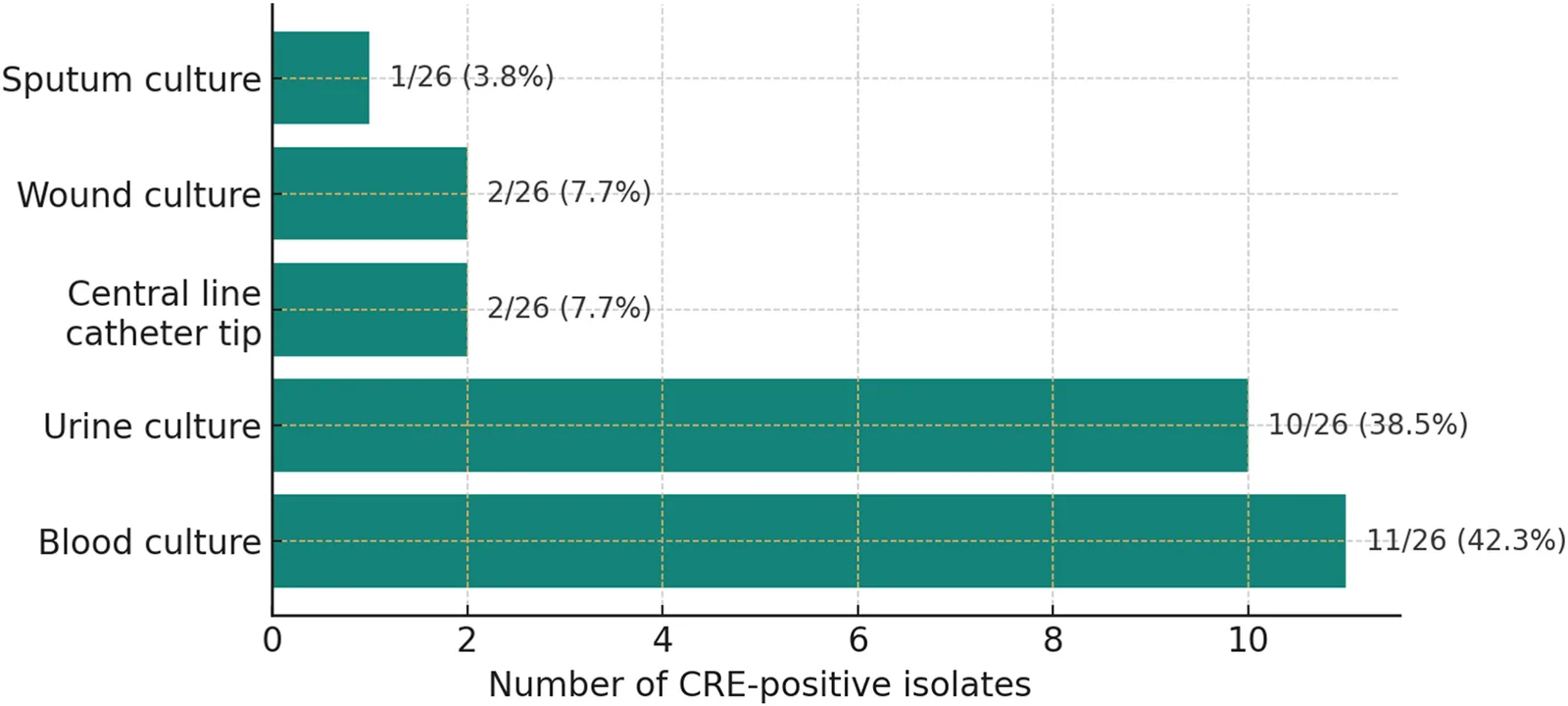
Distribution of CRE-positive isolates by specimen source.

Carbapenems are broad-spectrum beta-lactam antibiotics used to treat severe infections caused by MDR Enterobacterales. Their use as a therapeutic option of choice has lately been extended because of an increase in the prevalence of MDR Enterobacterales in many countries [1]. Concurrently, the increasing global burden of carbapenem-resistant Enterobacterales has become a major public health concern. This concern is due to the resistance profile of CRE, limited therapeutic options, adverse impacts on patient outcomes, and an associated increase in morbidity and mortality, especially in health care settings [2] (see Fig. 1, Fig. 2).

CRE emergence is mediated by the acquisition and expression of carbapenemase enzymes encoded by genes spanning three Ambler classes: KPC (*Klebsiella pneumoniae* carbapenemase; class A); IMP (Imipenemase), NDM-1 (New Delhi metallo-β-lactamase), and VIM (Verona integron-encoded metallo-β-lactamase; class B); and OXA-48-like (OXA: oxacillinase) carbapenemases (class D) [3]. Non-enzymatic mechanisms contributing to carbapenem resistance include loss or mutation of porin-encoding genes and the overexpression of efflux pump–encoding genes [4].

CRE isolates are predominantly represented by *Klebsiella pneumoniae*. On a global scale, KPC is the most common carbapenemase and is usually plasmid-mediated in *Klebsiella pneumoniae* [5]. In Saudi Arabia, however, the leading carbapenemases are OXA-48, followed by NDM [6].

KPC is a β-lactamase that hydrolyzes carbapenem antibiotics and can thereby contribute to treatment failure in severe infections. It is encoded by the blaKPC gene and is produced by several bacterial species belonging to the Enterobacterales group [7]. The main aim of this study was to determine the frequency with which Enterobacterales clinical isolates show resistance to carbapenems. It also aimed to describe them at the molecular level, with particular reference to resistance mechanisms and factors. It is important to understand the molecular nature of CRE infections.

## 2 Methods

This was a cross-sectional analytic study conducted at Prince Mohammed Bin Nasser Hospital, Jazan, Saudi Arabia. All clinically relevant data was collected prospectively from electronic medical records between June 2023 and April 2024.

**Inclusion criteria:** All isolates of Enterobacterales recovered from the clinical specimens of hospitalized patients between June 2023 and April 2024 were included.

**Exclusion criteria:** Isolates from outpatients or emergency department patients, carbapenemase-producing organism screening isolates, repeated isolates from the same patient at the same anatomical site during the same admission, and samples yielding carbapenem-producing organisms other than Enterobacterales were excluded. To minimize over-counting, a first-isolate-per-patient–species 14-day rule was applied: only the initial isolate of a given species for each patient within any 14-day period was included in the primary analysis.

Microbiological and molecular testing was performed in the Microbiology Department of Prince Mohammed Bin Nasser Hospital, Jazan. Clinical specimens, including blood, respiratory secretions, wound swabs, and urine, were processed according to standard microbiological procedures. Specimens were inoculated onto appropriate routine culture media, including blood agar, chocolate agar, MacConkey agar, and cystine–lactose–electrolyte-deficient (CLED) agar for urine specimens, and incubated at 35–37°C for 18–24 h. After incubation, suspected Enterobacterales colonies were subcultured to obtain pure isolates and identified to the species level using the VITEK® 2 Compact automated identification system (bioMérieux, Marcy-l'Étoile, France). No additional confirmatory method was used for species identification. Antimicrobial susceptibility testing was performed using the VITEK® 2 system, and the results were interpreted according to the Clinical and Laboratory Standards Institute (CLSI) M100, 33rd edition (2023). Carbapenem susceptibility was interpreted using the CLSI breakpoints for imipenem and meropenem (susceptible ≤1 μg/mL, intermediate = 2 μg/mL, and resistant ≥4 μg/mL). Isolates demonstrating phenotypic resistance to at least one of these carbapenems were classified as carbapenem-resistant Enterobacterales (CRE) and were subsequently analyzed using the Cepheid Xpert® Carba-R assay (Cepheid, Sunnyvale, CA, USA). The assay detects the major carbapenemase gene families blaIMP, blaKPC, blaNDM, blaOXA-48, and blaVIM.

**Statistical analysis:** All statistical calculations were done using R version 4.2.1 and SPSS version 28.0. The demographic information, such as sex, nationality, age, and age groups, was expressed in simple terms, where categorical information was expressed as how frequently a category occurred and the percent of each category. Prevalence refers to the proportion of cases with CRE.

The relationship of clinical risk factors with CRE isolation was assessed using Pearson's chi-square test when expected cell frequencies were at least 5, or Fisher's exact test otherwise. Odds ratios with 95% confidence intervals were calculated to quantify these associations. When one or more contingency-table cells contained zero events, an uncorrected odds ratio was not estimable and was reported as not calculated (NC) rather than as 0.00. The risk factors considered included intensive care unit admission, central venous catheterization, Foley catheterization, endotracheal intubation, diabetes, hypertension, hematologic malignancies, and solid tumors. Significance was set at 0.05. The assessment of CRE genes, such as NDM and OXA-48, was done, showing their co-occurrence in different specimen types.

## 3 Results

The study analyzed 260 Enterobacterales isolates obtained from 260 patients. In the cohort, the sex distribution was almost equal, with 131 of 260 patients being male (50.4%) and 129 of 260 being female (49.6%). Saudi nationals were 199 of 260 patients (76.5%), while 61 of 260 (23.5%) were non-Saudi nationals. The patients were divided among the age groups as follows: ≥ 60 years (119/260, 45.8%), adults 18-59 years (109/260, 41.9%), children 0-12 years old (24/260, 9.2%), and adolescents 13-17 years old (8/260, 3.1%). Their mean age was 55.6 years, with a median of 63 years.

Of the 260 isolates, 26 (10.0%) met the CRE definition by demonstrating phenotypic resistance to at least one of the two carbapenems tested; the remaining 234 isolates (90.0%) did not meet the CRE definition. The main CRE species included *Klebsiella pneumoniae* (approximately 81%), the *E. cloacae* complex (approximately 11%), *Escherichia coli* (approximately 4%), and *Klebsiella oxytoca* (approximately 4%). Of the carbapenemase-producing isolates, the majority were NDM-positive (approximately 58%), followed by those with OXA-48 and NDM (approximately 23%), and those with OXA-48 alone (approximately 19%). None had KPC, VIM, or IMP. CRE isolates were distributed almost equally between blood (11/26, 42.3%) and urine (10/26, 38.5%); the remaining five isolates (19.2%) were recovered from other specimen types (see Table 1).

In the bivariate analyses (Table 2), several device- and care-related variables showed strong quantitative associations with CRE isolation. Use of a central venous catheter was associated with nearly a sevenfold increased odds of CRE isolation (odds ratio [OR] 6.80, 95% confidence interval [CI] 2.84–16.27; χ^2^ = 23.834; p < 0.001; post-hoc power = 0.99). Foley catheterization was associated with more than a fourfold increased odds (OR 4.49, 95% CI 1.84–10.96; χ^2^ = 11.162; p = 0.004; power = 0.93). Critical care unit admission was also associated with higher odds of CRE (OR 2.83, 95% CI 1.22–6.55; χ^2^ = 7.821; p = 0.020), although post-hoc power was modest (0.69), reflecting limited event numbers.

**Table 1:** Patient demographics.

Characteristic	N (%)
**Age**	
*Child*	24 (9.2%)
*Adolescent*	8 (3.1%)
*Adult*	109 (41.9%)
*Older adult*	119 (45.8%)
*Mean (years)*	55.58
*Median (years)*	**63**
**Gender**	
Male	131 (50.4%)
Female	129 (49.6%)
**Nationality**	
Saudi	199 (76.5%)
Non-Saudi	61 (23.5%)

**Table 2:** Risk factors for acquisition of carbapenem-resistant Enterobacterales (CRE).

Risk factor	Chi-Square Value	P-value	OR^a^ (95% CI^b^)	Post Hoc Power
Critical care unit admission	7.821	0.02	2.83 (1.22-6.55)	0.69
Central venous line catheter	23.834	0.000	6.80 (2.84-16.27)	0.99
Foley catheter	11.162	0.004	4.49 (1.84-10.96)	0.93
Endotracheal intubation	4.711	0.095	2.24 (0.93-5.39)	0.42
Solid organ malignancy	0.101	0.951	NC	NA^c^
Hematological malignancy	5.246	0.073	NC	0.82
Hemoglobinopathy	0.628	0.731	NC	NA^c^
Diabetes mellitus	3.844	0.206	NC	NA^c^
Hypertension	3.16	0.146	NC	NA^c^

a OR: Odds ratio.

b CI: confidence interval.

c NA: not applicable. NC: not calculated because at least one contingency-table cell contained zero events; the uncorrected OR and 95% CI were therefore not estimable.

The other factors did not achieve conventional statistical significance. For endotracheal intubation, the OR was 2.24 (95% CI 0.93–5.39; χ^2^ = 4.711; p = 0.095; post-hoc power = 0.42). For hematologic malignancy, solid organ malignancy, hemoglobinopathy, diabetes, and hypertension, ORs were not calculated because zero cell counts precluded valid uncorrected estimation; these entries were therefore reported as NC rather than 0.00. Given that only 26 CRE cases were identified, these nonsignificant or non-estimable findings should be interpreted as imprecise and should not be interpreted as protective effects or evidence of no association.

## 4 Discussion

The prevalence found in this study is 10%, which is slightly higher than that reported by Al-Abdely et al. (2021) [8] as 8.6%. In contrast, studies conducted in other regions of Saudi Arabia show higher rates; for instance, Said et al. (2021) reported a prevalence of over 20% in Ha'il [9]. *Klebsiella pneumoniae* was the most prevailing CRE species, accounting for 81% of the total CRE isolates in our study. It is similar to the observations assessed by Al-Abdely et al. (2021), who reported the prevalence of this species in 90% of CRE isolates. *Klebsiella pneumoniae* remains the most common carbapenem-resistant pathogen worldwide [8]. There is a consistent pattern across Asia, Africa, and the Americas, driven mainly by the organism's remarkable ability to acquire and spread genes encoding for carbapenemases, including KPC, NDM, and OXA-48-like enzymes [10,11]. The strong association with nosocomial outbreaks and HAIs further solidifies its global predominance among carbapenem-resistant pathogens [12].

Among the currently circulating strains, NDM was the predominant carbapenemase gene detected (58%), followed by 23%, which harbored both OXA-48 and NDM, and 19%, which harbored OXA-48 alone. KPC, VIM, and IMP were not detected in these strains. Previous studies in Saudi Arabia reported OXA-48 in 58%, 81%, NDM in 20%, 42%, and both NDM and OXA-48 in 8%, 14%, respectively [8]. Al-Mohammadi et al. (2022) agreed with our results in having 45% NDM in carbapenem-resistant *Klebsiella pneumoniae* in Yemeni hospitals as reported in their study [13]. This regional alignment could be attributed to geographic proximity and population mobility across borders.

In our cohort, NDM-type carbapenemases predominated as the major resistance mechanism, which fits with the broader geographical pattern wherein NDM-producing Enterobacterales have become established throughout South Asia and the Middle East. In contrast, KPC producers constitute the main CRE burden in several settings in America and Europe–Latin America, including the United States, Brazil, and Greece, where they account for approximately three-quarters to nearly all CRE isolates [10]. OXA-48 and related enzymes are more frequently reported from North Africa and countries bordering the Mediterranean [10]. These observations support the observation that our local resistance profile fits into a regional NDM–OXA-48 axis rather than the KPC-dominated epidemiology seen elsewhere, and is likely influenced by region-specific clonal spread, antimicrobial use policies, and infection prevention capacity.

There was a similar age distribution in the multicentre Saudi study of Alraddadi et al., in 2022 as observed in our study, where the CRE infections tended to cluster among the older inpatients [14]. The clearest statistical signal in our analysis was for device- and care-related exposures. The review by Elhaj (2024) [15] and the meta-analysis by Singh et al. (2025) [16] both identified ICU stay, central lines, and urinary catheters as recurrent risk factors, observations that are in accordance with our findings. With few cases of CRE, particularly among populations with other health conditions, the absence of evidence should be interpreted with caution as an imprecise estimate, not as evidence for the absence of effect. Blood and urine accounted for comparable proportions of CRE isolates (42.3% [11/26] and 38.5% [10/26], respectively), differing by only one isolate; therefore, the study did not establish blood as a clearly predominant specimen source. The distribution nevertheless demonstrated both bloodstream and urinary presentations. Alraddadi et al. (2022) reported that nearly half of CRE infections (46.6%) were bloodstream infections [14]. Nassar et al. (2023) reported an even higher proportion of bacteremic presentations (53.4%), followed by urinary tract infections [17]. We did not systematically record patient-level outcomes such as length of stay, duration of ICU treatment, mortality, or treatment response.

The meta-analysis by Zhang et al. (2023) reported mortality exceeding 40% among ICU patients with CRE bacteremia, highlighting the potential severity of these infections [18].

## 5 Limitations

Because this was a single-center study, the findings may not be generalized to other settings or geographic regions. Also, by focusing on patients who had been hospitalized, community-acquired infections were excluded, thus potentially underestimating the true prevalence of CRE [19]. Our study characterizes the prevalence of CRE and the main types of carbapenemase genes, but data collection did not include information regarding patient outcomes like the length of hospital stay, admission to an ICU, and mortality rate. Future studies should include such outcomes to more accurately convey the burden of CRE and help shape strategies for infection control and antibiotic use.

## 6 Conclusion

Our study contributes to the growing body of research on the molecular aspects of CRE in Saudi Arabia. There are several related factors that contribute to the differing resistance rates of CRE in different regions. The effectiveness of Infection Control Practices, such as hand washing, as well as the management of devices in hospitals, is also an important factor. In addition, the facilities' ability to diagnose the resistant strain, including those with multiple carbapenemase genes, also plays an important role.

## CRediT authorship contribution statement

**Hanan Alshafie:** Conceptualization, Data curation, Supervision, Writing – original draft, Writing – review & editing. **Fathima Tarannum:** Writing – original draft, Writing – review & editing. **Mousa Ayyashi:** Data curation, Investigation, Methodology. **Nawal Yaqoub:** Formal analysis, Methodology. **Maryam Hazazi:** Data curation. **Sultan Masmali:** Data curation. **Durga Prasad Dommeti:** Data curation, Project administration. **Halima Babgi:** Data curation.

## Informed consent statement

Not applicable.

## Ethical approval

The study was approved by the Jazan Ethics committee (Approval No. 2354 on Sunday, June, 2023), SA.

## Data availability statement

The datasets generated and/or analyzed during the current study are not publicly available due to patient confidentiality and institutional data protection policies but are available from the corresponding author on reasonable request and with appropriate ethical approvals.

## IA assist

Artificial intelligence (AI) was used solely for language editing and formatting assistance. No AI tools were used for data analysis, interpretation, or content generation. The authors take full responsibility for the integrity and accuracy of the manuscript's content.

## Funding

This work was not funded by any institution.

## Declaration of competing interests

The author declares no conflicts of interest.
